# Endoscopic ultrasound-guided antegrade treatment using a novel nonslip short-length balloon catheter for hepaticojejunostomy anastomotic stricture

**DOI:** 10.1055/a-2559-4316

**Published:** 2025-03-28

**Authors:** Tadahisa Inoue, Rena Kitano, Tomoya Kitada, Kazumasa Sakamoto, Satoshi Kimoto, Jun Arai, Kiyoaki Ito

**Affiliations:** 1 12703 Department of Gastroenterology, Aichi Medical University, Nagakute, Japan

Balloon stricture dilation via balloon enteroscopy-assisted endoscopic retrograde cholangiopancreatography is the standard treatment for hepaticojejunostomy anastomotic stricture (HJAS) 1 , but accessing the anastomosis is often challenging. In such cases, an endoscopic ultrasound (EUS)-guided antegrade approach can be considered as an alternative.

Conventional balloons designed for papillary, or bile duct dilation are often too long for treating HJAS, which may lead to unnecessary dilation of the intrahepatic bile ducts. In contrast, short balloons are prone to slipping during inflation, presenting a greater challenge in the EUS-guided approach. This is because the position of the balloon cannot be directly visualized endoscopically, making precise positioning and adjustment during inflation more difficult.

We developed a novel dedicated balloon catheter ( Fig. 1 ), which was designed to address these challenges. This balloon is exceptionally short, measuring only 15 mm, and features an elastic band at its center. The band delays the expansion of the central portion during inflation, effectively preventing slippage 2 . Furthermore, the tapered tip is designed to enhance insertion and pushability, ensuring optimal performance when passing through the fistula and stricture.

**Fig. 1:**
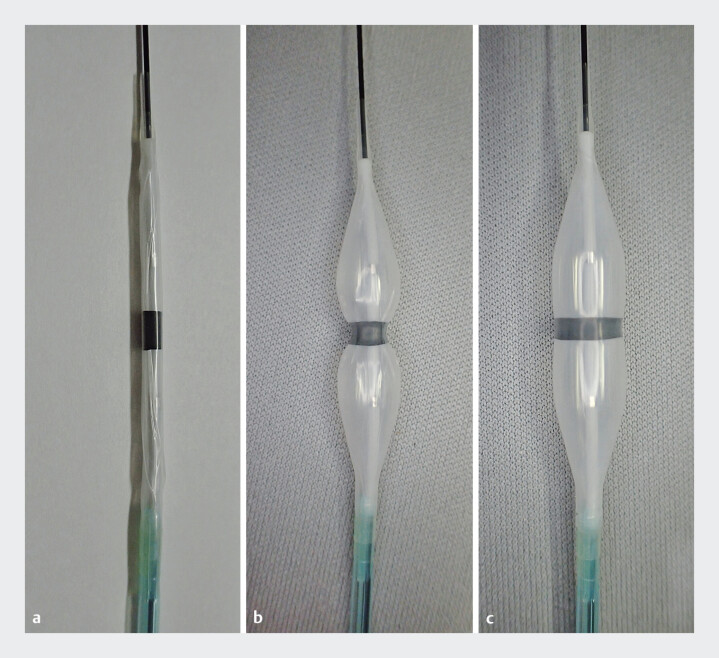
The novel dedicated balloon catheter (RIGEL; Japan Lifeline, Tokyo, Japan) measures 15 mm in length, shorter than conventional balloons (typically 30–50 mm). **a** The catheter features an elastic band in the middle. **b, c** The elastic band results in delayed expansion of the central portion, preventing slippage. The tapered tip is designed to enhance insertion and pushability, ensuring optimal performance when passing through the fistula and stricture.

An 86-year-old woman who had undergone hepaticojejunostomy with Roux-en-Y reconstruction developed obstructive jaundice caused by HJAS. A short-type single-balloon enteroscope could not reach the anastomosis, so a linear-array echoendoscope was used instead ( Video 1 ). The left intrahepatic bile duct was punctured from the stomach using a 19-G needle, and a 0.025-inch guidewire was inserted and advanced through the HJAS into the jejunum ( Fig. 2 **a** ). A standard catheter was inserted, and contrast medium was injected to confirm the HJAS ( Fig. 2 **b** ). Subsequently, the novel 8-mm-diameter balloon was introduced and positioned at the site of the HJAS ( Fig. 2 **c** ). The central portion of the balloon expanded with a controlled delay during inflation, allowing full expansion without slippage ( Fig. 2 **d** ). The stricture was successfully recanalized, resulting in good contrast flow from the intrahepatic bile ducts into the small intestine, with no adverse events.

**Fig. 2:**
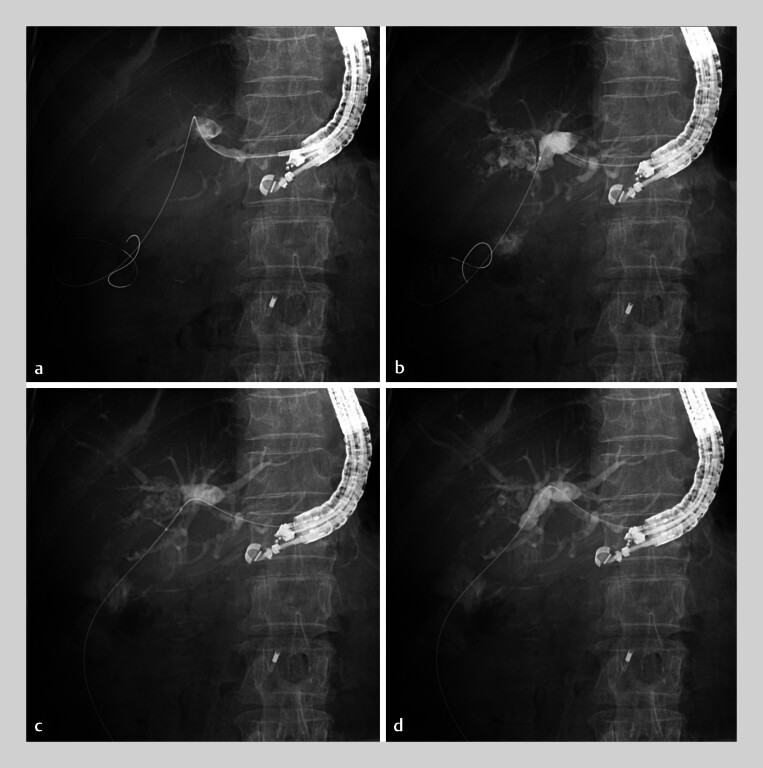
Fluoroscopic images. **a** The left intrahepatic bile duct was punctured from the stomach using a 19-G needle, and a 0.025-inch guidewire was inserted and advanced through the hepaticojejunostomy anastomotic stricture (HJAS) into the jejunum. **b** A standard catheter was inserted, and contrast medium was injected to confirm the HJAS. **c** Subsequently, the novel 8-mm-diameter balloon was introduced and positioned at the site of the HJAS. **d** The central portion of the balloon expanded with a controlled delay during inflation, allowing full expansion without slippage.

Endoscopic ultrasound-guided antegrade treatment using a novel nonslip short-length balloon catheter in a patient with hepaticojejunostomy anastomotic stricture.Video 1

Endoscopy_UCTN_Code_TTT_1AS_2AH

## Notes

Endoscopy E-Videos https://eref.thieme.de/e-videos
*E-Videos* is an open access online section of the journal *Endoscopy* , reporting on interesting cases and new techniques in gastroenterological endoscopy. All papers include a high-quality video and are published with a Creative Commons CC-BY license. Endoscopy E-Videos qualify for HINARI discounts and waivers and eligibility is automatically checked during the submission process. We grant 100% waivers to articles whose corresponding authors are based in Group A countries and 50% waivers to those who are based in Group B countries as classified by Research4Life (see: https://www.research4life.org/access/eligibility/ ). This section has its own submission website at https://mc.manuscriptcentral.com/e-videos .
